# Proteome profiling in IL-1β and VEGF-activated human umbilical vein endothelial cells delineates the interlink between inflammation and angiogenesis

**DOI:** 10.1371/journal.pone.0179065

**Published:** 2017-06-15

**Authors:** Thomas Mohr, Verena Haudek-Prinz, Astrid Slany, Johannes Grillari, Michael Micksche, Christopher Gerner

**Affiliations:** 1 Institute of Cancer Research, Department of Medicine I, Medical University of Vienna, Vienna, Austria; 2 ScienceConsult – DI Thomas Mohr KG, Guntramsdorf, Austria; 3 Department of Analytical Chemistry, Faculty of Chemistry, University of Vienna, Vienna, Austria; 4 Christian Doppler Laboratory on Biotechnology of Skin Aging, Department of Biotechnology, BOKU – University of Life Sciences, Vienna, Austria; 5 Evercyte GmbH, Vienna, Austria; Ottawa Hospital Research Institute, CANADA

## Abstract

Endothelial cells represent major effectors in inflammation and angiogenesis, processes that drive a multitude of pathological states such as atherosclerosis and cancer. Both inflammation and angiogenesis are interconnected with each other in the sense that many pro-inflammatory proteins possess proangiogenic properties and vice versa. To elucidate this interplay further, we present a comparative proteome study of inflammatory and angiogenic activated endothelial cells. HUVEC were stimulated with interleukin 1-β and VEGF, respectively. Cultured primary cells were fractionated into secreted, cytoplasmic and nuclear protein fractions and processed for subsequent LC-MS/MS analysis. Obtained protein profiles were filtered for fraction-specific proteins to address potential cross fractional contamination, subjected to comparative computational biology analysis (GO-Term enrichment analysis, weighted gene co-expression analysis) and compared to published mRNA profiles of IL-1β respectively VEGF stimulated HUVEC. GO Term enrichment analysis and comparative pathway analysis revealed features such as NOD and NfkB signaling for inflammatory activated HUVEC and VEGF and ErB signaling for VEGF-activated HUVEC with potential crosstalk via map kinases MAP2K2. Weighted protein co-expression network analysis revealed several potential hub genes so far not associated with driver function in inflammation or angiogenesis such as HSPG2, ANXA3, and GPI. “Classical” inflammation or angiogenesis markers such as IL6, CXCL8 or CST1 were found in a less central position within the co-expression networks. In conclusion, this study reports a framework for the computational biology based analysis of proteomics data applied to cytoplasmic, nucleic and extracellular fractions of quiescent, inflammatory and angiogenic activated HUVEC. Novel potential hub genes relevant for these processes were successfully identified.

## Introduction

The endothelium is a monolayer of cells covering the inner surface of blood vessels, lymphatic vessels, and the heart. In adults, it consists of approximately 1x10^13^ cells, weighs approximately 1kg and is a major metabolic organ with vital secretory, synthetic, metabolic, and immunologic functions [1]. It plays a key role in the generation and regulation of many physiological processes such as coagulation, hemostasis, and angiogenesis as well as pathophysiological processes such as atherosclerosis, cancerous metastasis dissemination, and inflammation [2]. Dysregulated inflammation and angiogenesis are major factors contributing to a wide array of diseases including cardiovascular diseases (CVD) and cancer [3,4].

Acute inflammation is a complex process including the control of tissue damage after pathogenic, traumatic or toxic injury that is tightly coordinated by pro- and anti-inflammatory molecules regulating chemotaxis, migration and cell activation. It is characterized by a rapid recruitment and activation of leukocytes such as neutrophils, eosinophils and natural killer cells followed by generation of reactive oxygen species and infiltration of leukocytes into the inflamed area to remove remaining pathogens [5]. Since acute inflammation requires a continuous stimulus to be sustained, it will resolve quickly due to the short half-life of inflammatory mediators once this stimulus is removed. While acute inflammation serves as a protective mechanism, chronic inflammation causes substantial tissue damage and may promote cancer development [6]. Chronic inflammation plays a crucial role in driving the development of atherothrombosis which is responsible for approximately 80% of all sudden cardiovascular deaths [7].

In this situation angiogenesis and inflammation act as mutual amplifiers by continuous recruitment and activation of pro-inflammatory cells which in turn induce angiogenesis by secretion of a plethora of pro-angiogenic factors [8]. Whereas the role of inflammatory cells in this process is quite well understood, the contribution of endothelial cells is less clear. It is known that endothelial cells are major drivers for initiation and control of inflammatory processes. Stimulation of endothelial cells with pro-inflammatory cytokines such as IL-1β leads to an activation of the NFκB pathway via linkage of the respective receptors to the inhibitor of the IκB-kinase complex. Consequently, NFκB subunits translocate as transcription factors into the nucleus to trigger a response program encompassing a large number of cytokines, chemokines and adhesion molecules involved in various aspects of the inflammatory response [4]. Many IL-1β induced proteins display a potent pro-angiogenic activity. IL6 triggers angiogenesis [9]. CXCL8 is known to upregulate VEGF and VEGFR2 expression in endothelial cells, thus triggering autocrine angiogenic activation [10].

VEGF is the primary mediator of both physiological and pathological angiogenesis. It is typically induced in tissues with low oxygen pressure and sensed by endothelial cells of nearby vessels. Upon stimulation with VEGF a gene expression profile characterized by genes related to cell survival, growth, migration, invasion into the surrounding tissue and formation of tube-like structures is induced in endothelial cells [11]. Somewhat surprisingly, the overlap between mRNA expression profiles of IL-1β and VEGF is relatively large (63%) [12]. Genes induced by both VEGF and IL-1β encompass major players of inflammatory activation such as CXCL1, CXCL3, CCL2, and CXCL8. Especially CXCL8 is known to promote activation and recruitment of macrophages and monocytes which is a prerequisite for the switch from acute to chronic inflammation [13]. Taken together, this points towards a regulatory role of VEGF in not only in angiogenesis but also in inflammation, mediated via PLC-γ and NFAT pathways rather than NF-κB [14].

Short-term treatment of endothelial cells with VEGF results in the upregulation of several pro-inflammatory cytokines. However, this upregulation remains comparatively weak compared to stimulation with IL-1β. Whereas IL-1β induced mRNA expression persists for several hours, VEGF-induced expression of this pro-inflammatory gene expression profile is transient and ceases almost entirely after 6 hours incubation [12]. Analysis of the transcriptome can only provide a list of potentially expressed proteins, whereas proteomic analysis reveals proteins that are actually present, thus resulting in a much more accurate picture of physiological and pathological cellular processes. The extent to which this pro-inflammatory mRNA profile translates into persistent protein expression after prolonged stimulation with VEGF remains unclear, therefore the investigation of specific protein expression patterns and the understanding of their relation to cellular mechanisms are essential for the successful identification of biologically relevant mechanisms or therapeutic targets.

Our research strategy, therefore, is the systematic analysis of primary human cells to investigate cell type or cell state specific protein expression patterns. A highly standardized protocol ensures that protein profiles derived from different human cell types are compatible and can be compared and combined with each other [15–19]. The in-house developed GPDE database contains protein profiles derived from more than 20 different human cell types and allows the combination of two or more protein profiles using sequential algebraic operations such as union, intersection and relative complement [20]. State of the art enrichment and network analysis methods allow us to put the obtained protein expression profiles into broader biologic context.

Various attempts have been made to investigate the protein profiles of both the cytoplasm and the secretome of EC [21–25]. However, these studies either focus on singular aspects of endothelial activation or investigate only the cellular content of endothelial cells. Recently Slany et al. investigated the proteome profile of inflammatory activated HUVEC and normal human dermal fibroblasts [25]. This study presents the first comparative proteome map of inflammatory (IL-1β) versus angiogenic (VEGF) activated endothelial cells fractionated by cellular location (cytoplasm, nucleus and extracellular space). We identified a total of 1575 proteins in the cytoplasm, 618 proteins in the secretome and 802 proteins the nuclei. Using GO-Term and Pathway enrichment analysis, we were able to assess persistent overlaps in pathways and biologic function between IL-1β and VEGF-activated cells. Application of count based protein abundance estimation and protein-coexpression networks for GO-Term defined biologic functions are presented and investigated with regard to their preservation in quiescent, IL-1β, and VEGF stimulated HUVEC.

## Materials and methods

### Cell culture

HUVEC were purchased from Lonza (Verviers, Belgium) and cultured in tissue culture flasks (Corning, Lowell, MA, USA) coated with 0.5 μg/cm^2^ human fibronectin (HFN, Millipore, Billerica, MA, USA) in complete medium (CM) consisting of Endothelial Basal Medium (EBM-2MV, Lonza, Basel, Switzerland) supplemented with Single Quot Bullet Kits (Lonza) according to the instructions of the manufacturer and 10% Fetal Calf Serum (FCS, PAA Laboratories, Pasching, Austria). Cells were used for experiments up to passage number 5. EBM-2MV supplemented with hydrocortisone and ascorbic acid but without serum or growth factors was used to starve cells.

### Cell stimulation

Semiconfluent cultures were washed twice with PBS and incubated for 24h in EBM-2(MV) supplemented with hydrocortisone and ascorbic acid (BM). The medium was changed, and cells were stimulated for further 24h with BM containing either 10ng/mL hrIL-1β (R&D) or 10ng/mL VEGF (Lonza, Verviers, Belgium). Untreated cells served as control. Concentrations of stimulants were chosen in accordance to dose response curves provided by the manufacturer and preliminary experiments (growth of HUVEC for VEGF and induction of pro-inflammatory surface proteins CD62e for IL-1β). Stimulation time was chosen to reflect the accumulation of specific proteins in the extracellular fraction.

### Functional assays

#### Immunofluorescence staining for IL-6 and CSF2

For immunofluorescence staining, Cells were harvested by trypsin detachment, seeded into HFN coated chamber slides at a density of 15,000 cells/cm^2^ and stimulated as described above. Cells were fixed for 12 minutes at -20°C in acetone/methanol 1:1 and blocked (30' incubation with PBS containing 1% FCS). Cells were incubated for one hour with antibody against IL-6 and CSF2 (abcam, Cambridge, UK, ab9324 and ab9693, dilution 1:200 in PBS-FCS), washed three times with PBS-FCS, stained for 1 hour with FITC labeled goat anti-mouse IgG (Sigma-Aldrich, St. Louis, MI, USA, diluted 1:100 in PBS-FCS) and embedded in Vectrashield hardcover (Vector Labs, Burlingame, CA, US). Micrographs were taken using a Zeiss Confocal microscope.

#### Quantification of IL-6 and IL-8 expression by ELISA

Cells were harvested by trypsin detachment, seeded into HFN coated 24 well plates at a density of 10.000 cells/cm^2^ and stimulated as described above. The supernatant was harvested, and expression of IL-6 and IL-8 was determined using Quantikine Kits (R&D Biosciences) according to the instructions of the manufacturer. Results are measured as optical density at 450nm with a reference wavelength of 620nm and expressed as %control.

#### Tube formation assay

Tube formation assays were carried out as described previously [26] with minor changes. Endothelial cells were starved for 24hours in (BM), harvested by trypsin detachment, seeded at a density of 5000 cells/well onto matrigel-coated μ-angiogenesis slides (Ibidi, Martinsried, Germany), and incubated in BM with or without 10ng/mL VEGF. After a 20 h incubation, micrographs were taken at 4fold magnification using a Nikon Eclipse Ti and a Nikon Digital Sight DS-Fi1C camera. Tube formation (total network length in pixel) was quantified using the angiogenesis analyzer plugin for ImageJ [27].

### Protein isolation and sub-cellular fractionation

Cells (10 donors for the secretome, 7 for the cytoplasm and 4 for the nucleic fraction) were stimulated as described above and proteins from were isolated as described by Gundacker et. al. [16]. The culture supernatant was sterile filtered and precipitated by overnight at -20°C by ethanol precipitation. The entire workflow was done at 4°C, with centrifugation steps for 5 mins at3500rpm. For the cytosolic fraction, cells were lysed in hypotonic lysis buffer (10 mM HEPES/NaOH, pH 7.4, 0.25 M sucrose, 10 mM NaCl, 3 mM MgCl2, 0,5% Triton X-100) and pressed through a 23g syringe. The cytoplasmic fraction was separated from nuclei by centrifugation and precipitated as described above. The pellet was resuspended with 100 mM Tris/HCl pH 7.4, 1 mM EDTA pH 7.5, 500 mM NaCl and incubated for 10 minutes followed by 1:10 dilution and 15 mins incubation with 10 mM Tris/HCl pH 7.4, 1 mM EDTA pH 7.5, 0.5% NP-40. After centrifugation, proteins of the supernatant were precipitated as described above. Protein precipitates were dissolved in sample buffer (7.5 M urea, 1.5 M thiourea, 4% CHAPS, 0.05% SDS, 100 mM DDT). All buffers were supplemented with protease inhibitors: PMSF (1 mM), aprotinin, leupeptin and pepstatin A (each at 1 μg/mL).

### 1D-PAGE and tryptic digest for subsequent shotgun analysis and silver staining

1D PAGE, silver staining, tryptic digest, and mass spectrometry analysis was performed as described previously [17]. Briefly, protein fractions were loaded on 12% polyacrylamide gels and electrophoresis was performed until the complete separation of a pre-stained molecular marker (Dual Color, Biorad, Hercules, CA) was visible. Gels were fixed with 50% methanol/10% acetic acid, washed and sensitized with 0.02% Na2S2O3. The gels were stained with ice cold 0.1% AgNO3 for 20 min, rinsed with bi-distilled water and developed with 3% Na2CO3/0.05% formaldehyde. Tryptic digest was carried out as in-gel digest, and Nano Flow-LC-MS/MS was carried out using HPLC-Chip technology (Agilent) equipped with a 40 nL Zorbax 300SB-C18 trapping column and a 75μm x 150mm Zorbax 300SB-C18 separation column. Peptide identification was performed by MS/MS fragmentation analysis with an ion trap mass spectrometer (XCT-Ultra, Agilent) equipped with an orthogonal nanospray ion source as described previously [28].

### Analysis of mass spectrometry data

MS/MS data were interpreted by the Spectrum Mill MS Proteomics Workbench software (Version A.03.03, Agilent) and searched against the SwissProt database for human proteins (version 14.3 containing 20,328 entries) allowing for a precursor mass deviation of 1.5 Da, a product mass tolerance of 0.7 Da, a minimum matched peak intensity (%SPI) of 70% and carbamidomethylation of cysteines was set as fixed modification. For further modifications only oxidized methionine was considered. The listed peptides were identified with scores calculated from sequence tag lengths and mass deviations.

### Construction of protein lists

For the generation of protein lists for the respective fractions only peptides scoring higher than 9.0 (FDR<0.059, as determined by searches against the corresponding reverse SwissProt Database) were considered for protein identification. MS/MS runs yielding low total peptide counts were excluded from further analysis. Proteins with at least one unique peptide scoring equal or higher than 13.0 (FDR<0.0021) were accepted as positively identified. Only proteins identified by at least two peptides over all experiments were used for further analysis. Mass spectrometry data were organized by the GPDE database developed at our laboratory [20]. Identification details for all presently identified proteins including all identified peptides, sequence coverages, peptide scores and MS2 spectra are fully accessible via PRIDE database, accession number PRD000053 (http://www.ebi.ac.uk/pride/archive/projects/PRD000053).

### Minimization of bias due to cross fraction contamination

Potential contamination between the cytoplasm and the nucleic fraction was assessed using MAPK1, 11, 12, 13 and 14, and PARP1 expression which have been used previously to demonstrate fraction purity [29,30]. To further minimize potential bias due to cross-fraction contamination a filtering strategy based on GO-Term annotation was applied. Within the cytoplasm fraction, only proteins implicitly or explicitly annotated with GO-Terms GO:0005737 (cytoplasm), GO:0005886 (plasma membrane), GO:0005576 (extracellular region), GO:0031012 (extracellular matrix) and GO:0043230 (extracellular organelle) were considered for further analysis. For the nuclear fraction only proteins annotated with GO-Terms GO:0005634 (nucleus) and for the extracellular fraction proteins only annotated with GO Terms GO:0005576 (extracellular region), GO:0031012 (extracellular matrix) and GO:0043230 (extracellular organelle) were considered for further analysis.

### Biological context of fraction-specific proteins

To put fraction-specific proteins into a biological context, we analyzed the GO-term enrichment (biological process) and pathway enrichment (pathway databases) using the Cytoscape plugin ClueGO in combination with CluePedia [31,32]. First proteins detected in untreated HUVEC were subtracted from the protein lists of the respective treatment (IL-1β and VEGFA). The resulting protein lists were used as input for comparative analysis between networks in ClueGO. For analysis of the cytoplasm fraction, the REACTOME and KEGG database were used due to their general coverage of pathways [33,34]. Since the nucleic and the extracellular fraction contain only target proteins of pathways but not their intermediaries gene ontology biological process database was used [35]. P-values were corrected according to Benjamini-Hochberg [36]. To increase the power of the analysis levels below 4 (unspecific terms) and above 12 (extremely specific terms) were excluded from GO-Term analysis.

### Weighted protein co-expression network analysis (WPCNA)

#### Estimation of protein abundance and fold change

The analysis of protein quantification data based on peptide counts remains a challenge. Similarities to tag count based mRNA technologies led us to employ an overdispersed Poisson model combined with empirical Bayes methods to estimate protein abundance and fold change [37,38]. Count data were filtered for detected proteins as described above and loaded into R version 3.2.4. Protein abundance was estimated by calculating peptide counts normalized to counts per million (CPM) using the edgeR package, log2 fold change was estimated based on variance stabilized average log2 CPM values [39].

#### Coexpression analysis and determination of potential key drivers

WGCNA has been designed to globally analyze microarray data across several tissues, treatments or disease conditions. It aims at robust identification gene co-expression correlation by constructing global gene networks based on co-expression. Although this method has been developed for microarray data, it has been successfully applied to peptide count based proteomic data [40]. For each treatment in each fraction, an unsigned weighted correlation network was calculated using an intramodular connectivity of greater than 0.75 as a criterion for potential key drivers [41]. To meet the criterion for scale-free topology, a β-value of 6 was chosen. Modules were constructed using inflammation and angiogenesis relevant GO-terms. A protein was considered to be part of a module if it was either explicitly or implicitly annotated with the respective GO-term (S3 Table).

### Inspection of the interactome neighborhood

The pathway commons interactome (http://www.pathwaycommons.org) neighborhood of potential hub genes was investigated using the parameters canonical gene name and “neighborhood” [42].

### Reanalysis of mRNA data

RNA data of IL-1β and VEGF stimulated HUVEC (E-GEOD-10778) were downloaded into R from ArrayExpress using the ArrayExpress package [43], and RMA normalized using oligo [44]. Results are presented as log fold change.

## Results and discussion

### Functionality of HUVEC

**Fig 1:**
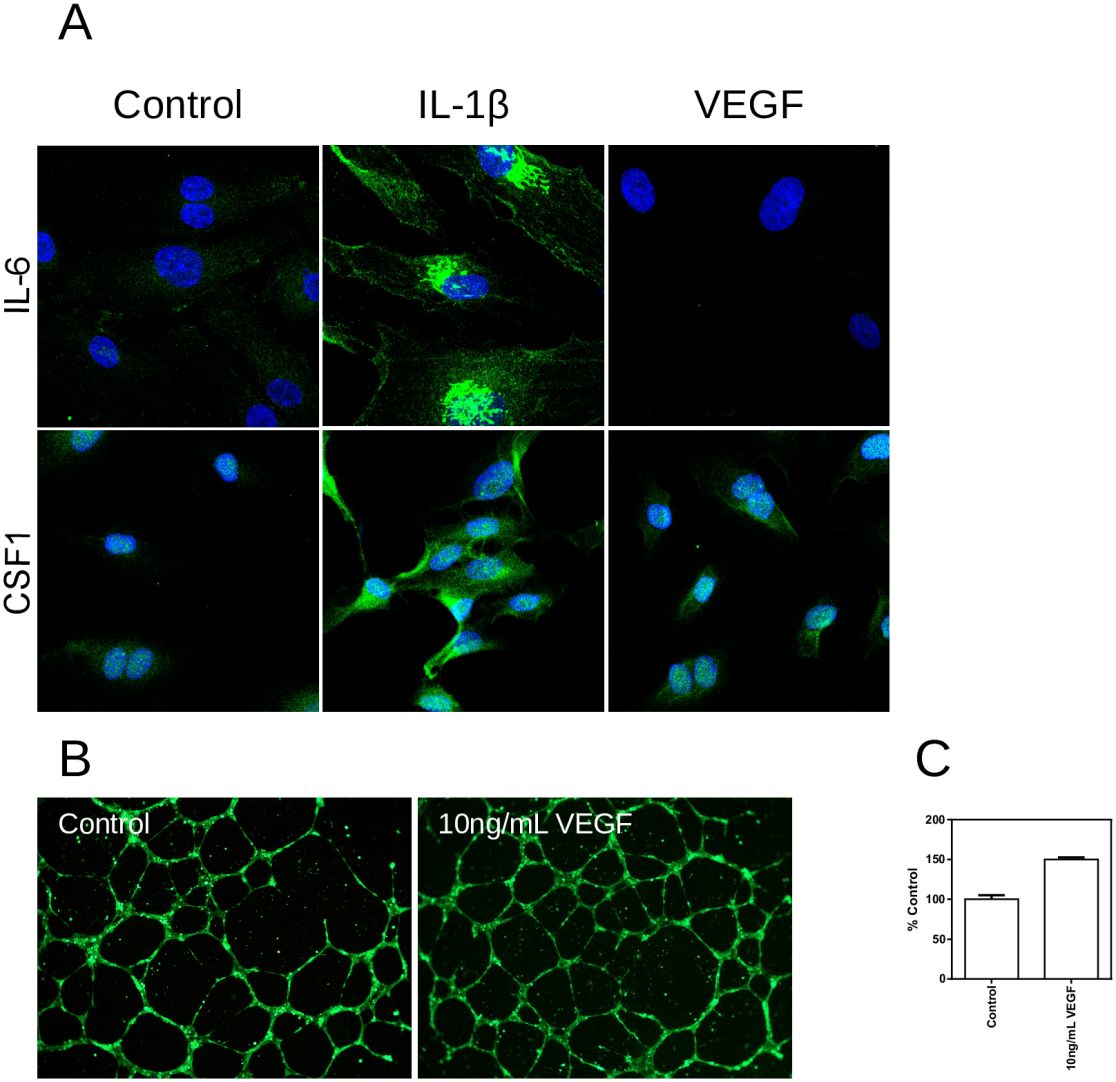
Immunofluorescence and tube formation in untreated HUVEC, HUVEC treated with 10ng/mL. IL-1β and HUVEC treated with 10ng/mL VEGF. (A) Immunofluorescence staining for IL-6 and CSF-1. (B) Calcein stained tubes of HUVEC treated with 10ng/mL VEGF (right) and the untreated control (left). (C) Quantification of tube formation using the angiogenesis plugin of ImageJ. Tube length was determined as pixels per tube, data are expressed as %control.

HUVEC displayed typical cobblestone morphology and stained positive (greater than 90%, data not shown) for CD31 and negative for CD90 as well as small muscle cell actin (less than 5%, data not shown) in flow cytometry. Treatment with 10ng/mL IL-1β resulted in an upregulation of IL-6. Treatment with IL-1β or 10ng/mL VEGF resulted in an upregulation of CSF1 (Fig 1a). Treatment with VEGF leads to increased tube formation to 150% of the control (Fig 1b). Taken together these data demonstrate the functionality of the chosen model system with regard to key endothelial functions [45].

### Proteome analysis

#### Preparation of protein extracts and estimation of cross-fraction contamination

In order to then investigate the interlink between inflammation and angiogenesis, we comparatively analyzed protein expression profiles of quiescent HUVEC, IL-1β and VEGF-treated HUVEC (10 donors for the extracellular fraction, 4 donors for the nuclear fraction and 7 donors for the cytoplasm fraction). The selection of these stimuli was based on the fact that IL-1β represents a main trigger of inflammation, whereas VEGF is the key initiator of angiogenesis. Potential cross-contamination between the nuclear (11 samples from 4 donors, 4 samples for quiescent HUVEC, 3 samples for IL-1β and 4 samples for VEGF-treated HUVEC) and the cytoplasmic fraction (18 samples from 7 donors, 6, 5 and 7 samples for the respective treatments) was assessed using peptide hit analysis for nuclear versus cytoplasmic marker proteins.

**Fig 2:**
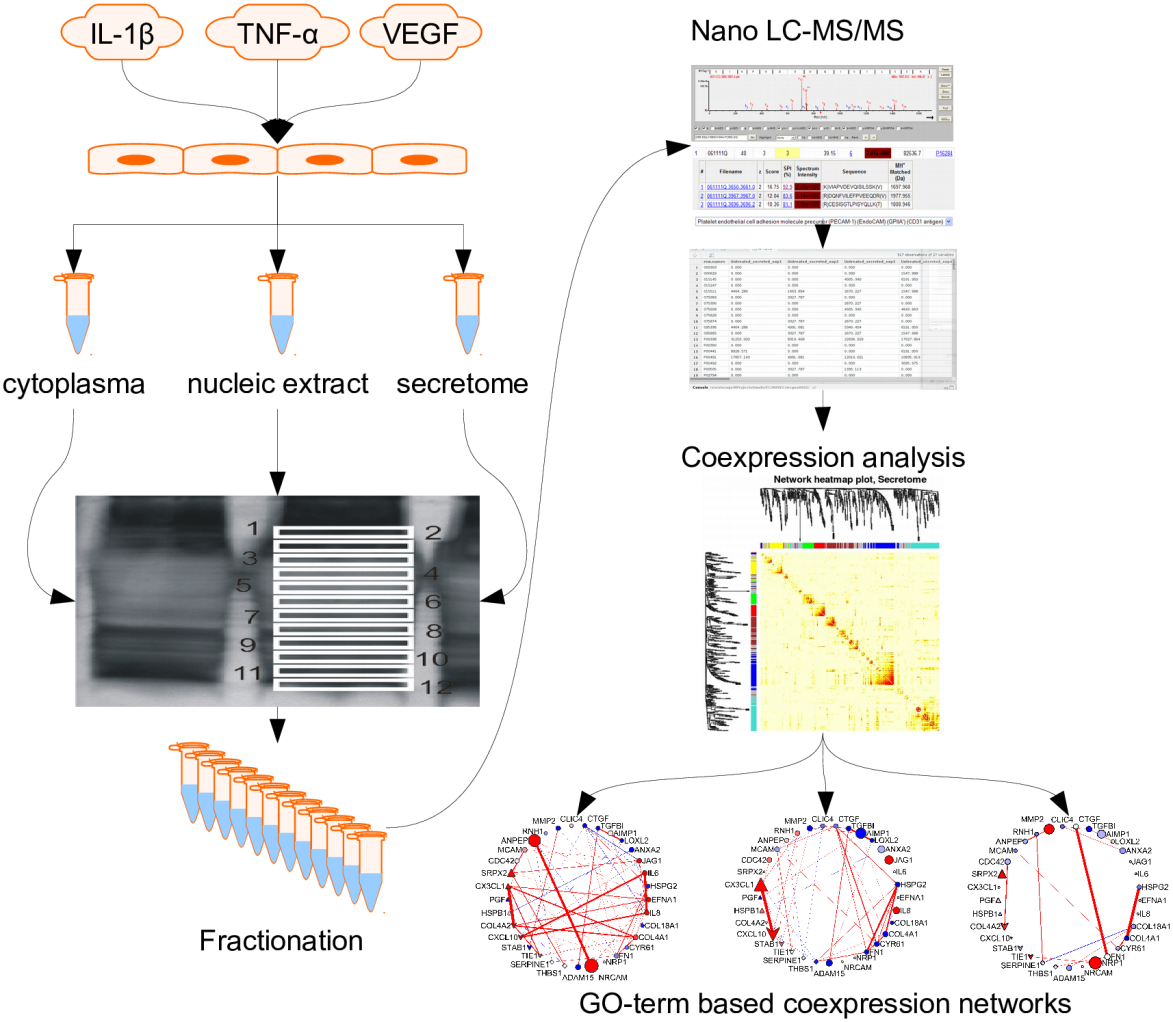
Experimental design of the study. HUVEC were left untreated or stimulated for 24 hours with 10ng/mL IL-1β β or 10ng/mL VEGF. Proteins from three cellular fractions (cytoplasm, nuclei and the extracellular fraction) were isolated and subjected to nano-LC-MS/MS. Results were analyzed using term enrichment and go-term based co-expression analysis methods.

After stimulation for 24 hrs, fractionation and nano-LC-MS/MS analysis (Fig 2) 1737 proteins were identified in the cytoplasm preparation, 1345 proteins in the nucleic fraction and 687 proteins in the cell supernatant. In these sets, previously used cytoplasm markers MAPK11, 12, 13, and 14 [29] could not be detected in the nucleic fraction, whereas nucleic marker PARP1 [30] was not detected in the cytoplasmic fraction, thus demonstrating the validity of the fractionation method.

GO term filtering for subcellular location resulted in 1575 out of 1737 (90,6%) (cytoplasm), 802 out of 1345 (59,6%) (nuclei), and 616 out of 687 (89,7%) (extracellular fraction) proteins being considered for further analysis (S1 Table). It is not entirely clear whether the comparatively high percentage of excluded proteins within the nucleic fraction is due to cross fraction contamination or due to misannotation of shuttling proteins. However, the fact that a highly expressed cytoplasmic protein (MAPK1) was not detectable in the nucleic fraction leads us to the conclusion that the proteins excluded from the nucleic fraction are predominantly proteins shuttling into the nucleus, thus lacking annotation with nucleus related subcellular locations. However, to improve robustness, all further analyses include only proteins annotated with the respective subcellular locations.

**Fig 3:**
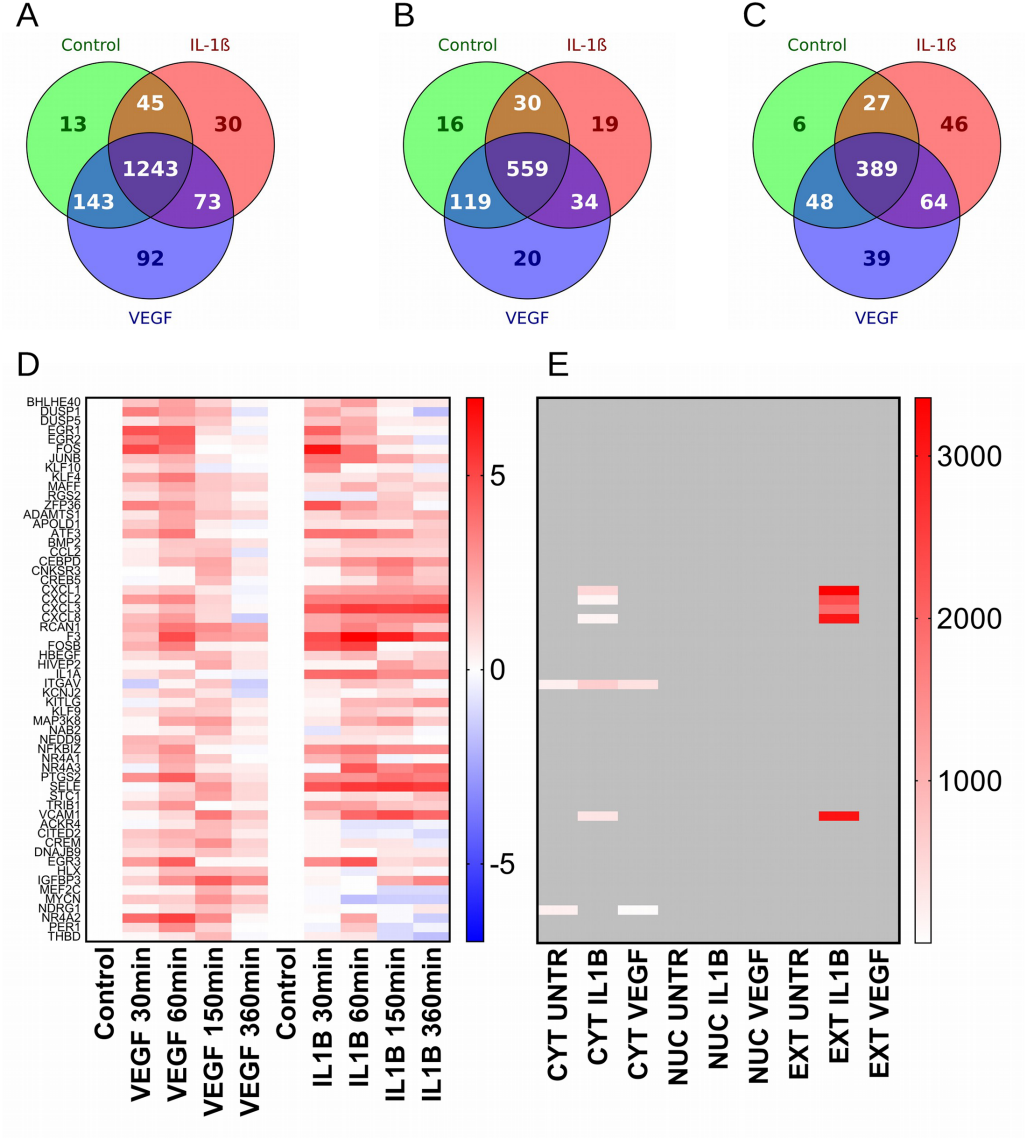
Venn diagrams and heat maps of the proteins detected HUVEC. Venn diagrams are displayed for untreated HUVEC (green circles), HUVEC treated with 10ng/mL IL-1β (red circles) and HUVEC treated with 10ng/mL VEGF (blue circles). (A) cytoplasm fraction, (B) nucleic fraction, (C) extracellular fraction, (D) reanalysis of E-GEOD-10778 (IL-1β and VEGF stimulated HUVEC, mRNA of genes regulated by IL-1β or VEGF stimulation, red = upregulated, blue = downregulated) and (E) corresponding protein expression (red = expression level, grey = not detected).

In the cytoplasm fraction, 1243 proteins were found to be expressed in all three treatments. Untreated HUVEC expressed 13 proteins exclusively, whereas IL-1β treatment resulted in the induction of 30 proteins, VEGF treatment resulted in the induction of 92 proteins. IL-1β and VEGF-treated HUVEC had a considerable overlap of 73 induced proteins whereas the overlap between treatments and the untreated control was 45 proteins for IL-1β and 143 proteins for VEGF (Fig 3a).

In the nucleic fraction, 559 proteins were found to be expressed in all three treatments. Untreated HUVEC expressed 16 proteins exclusively, whereas IL-1β treatment resulted in the induction of 19 proteins, VEGF treatment resulted in the induction of 20 proteins. IL-1β and VEGF-treated HUVEC had an overlap of 34 proteins whereas the overlap between treatments and the untreated control was 30 proteins for IL-1β and 119 proteins for VEGF (Fig 3b).

In the extracellular fraction (10 donors, 7 samples for untreated HUVEC, 6 samples for IL-1β and 10 samples for VEGF treatment), 389 proteins were commonly expressed. Untreated HUVEC expressed 6 proteins exclusively, whereas IL-1β treatment resulted in the induction of 27 proteins, VEGF treatment resulted in the induction of 39 proteins. IL-1β and VEGF-treated HUVEC had an overlap of 64 induced proteins whereas the overlap between treatments and the untreated control was 27 proteins for IL-1β and 48 proteins for VEGF (Fig 3c). S1 Table shows a list of detected proteins and their normalized abundance values.

Interestingly, the overlap between VEGF and IL-1β induced proteins was far lesser than it has been reported on mRNA Level [12]. The overlap between VEGF and IL-1β constituted 78% of the induced genes. Only 8% respective 22% of upregulated genes were selectively induced by IL-1β or VEGF [12]. We observed in all three fractions roughly half the overlap (37% for the cytoplasm, 46% for the nuclei and 42% for the extracellular fraction). For IL-1β, the percentage of specifically induced proteins was much higher (31% for the cytoplasm, 19% for the nuclei and 46% for the extracellular fraction) and for stimulation with VEGF the percentage of specifically induced proteins was comparable to RNA data only in the nuclei (27%), but not in the cytoplasm or the extracellular space (27% and 26% respectively).

After 24 hours incubation, we detected only 8 proteins associated with VEGF and/or IL-1β induced genes as shown in mRNA data. Only ITGAV was detected in IL-1β as well as VEGF stimulated HUVEC. Key pro-inflammatory proteins (CXCL1,2,3,6 and 8 and VCAM1) that have been found to be upregulated on mRNA level by both IL-1β and VEGF could only be detected in the cytoplasm or the extracellular space of IL-1β-treated HUVEC but not in VEGF-treated HUVEC (Fig 3e). The low overlap between mRNA and protein data might be explained by sensitivity differences between microarray technology and LC-MS/MS technology as well as by differential regulation on mRNA and protein level. However, the key pro-inflammatory proteins CXCL1,2,3,6 and 8, as well as VCAM1 were detected with a solid signal in the secretome of IL-1β stimulated HUVEC. The absence of all 6 proteins in the secretome of VEGF-treated HUVEC leads us to the conclusion that a possible induction of these proteins by VEGF is very short lived or below the detection limit.

A closer look at the time-response heat maps reveals that upregulation of all these genes is transient in VEGF-treated HUVEC with logFC levels near or below zero after 6 hours incubation. In contrast, mRNA expression of these genes in IL-1β-treated HUVEC remains elevated (Fig 3d) [12]. From these observations, we draw three conclusions. First, VEGF pre-programs endothelial cells in the direction of an inflammatory response. Second, for the VEGF-initiated pro-inflammatory program to be executed, co-stimulation is necessary. Finally, the IL-1β and to an even greater extent, the VEGF response involves extensive posttranslational regulation resulting in a diversification of protein expression at a later stage.

### Term enrichment analysis of proteins induced by Interleukin-1 β or VEGF

#### Cytoplasmic fraction

**Fig 4:**
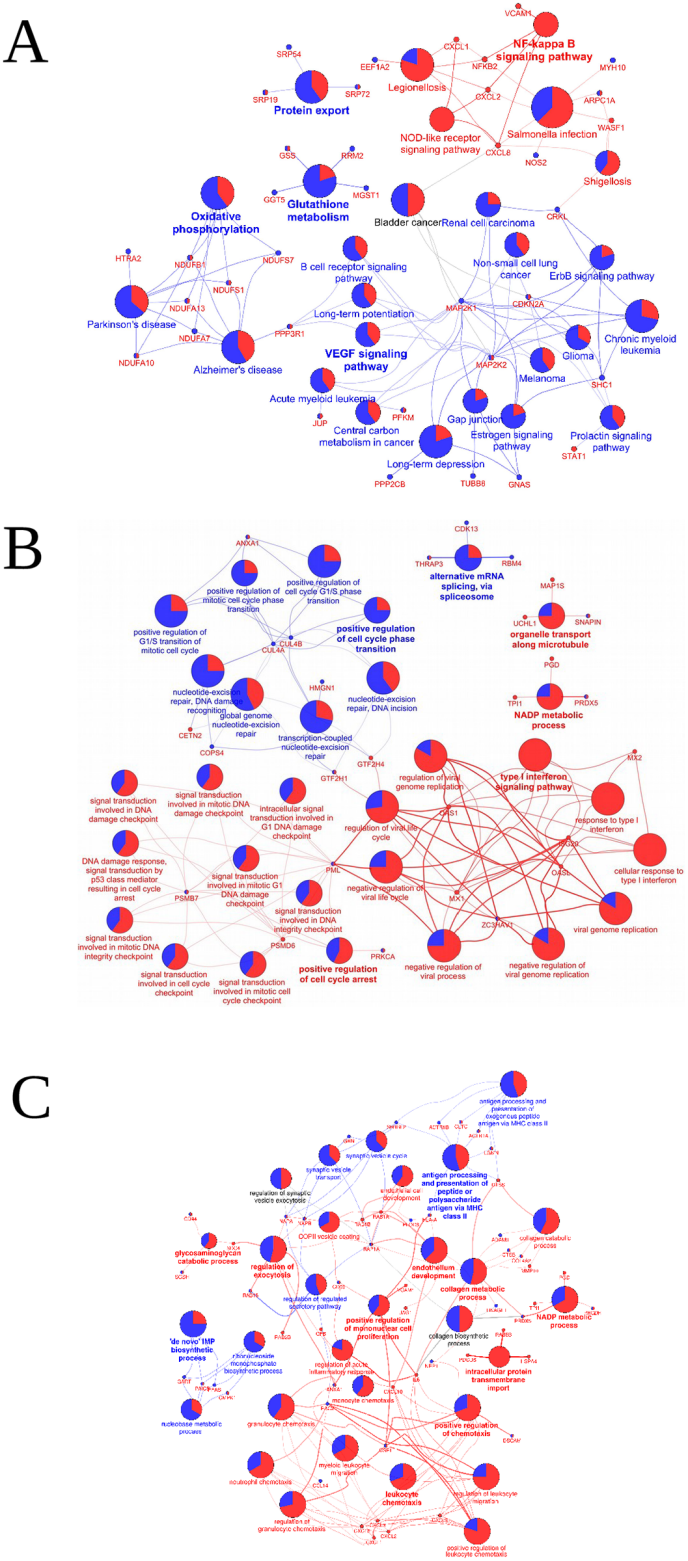
ClueGO analysis of the fractions of HUVEC treated with 10ng/mL IL-1β and HUVEC treated with 10ng/mL VEGF with untreated HUVEC as control. (A) cytoplasm fraction, (B) nucleic compartment, (C) extracellular fraction. Color depicts the fraction of induced proteins in IL-1β-treated HUVEC (red) and VEGF-treated HUVEC (blue). Node size depicts the p-value of the pathway. A summary of the analysis is provided in S2 Table.

To analyze the biological context of IL-1β or VEGF-induced cytoplasmic proteins, we constructed protein lists defined by subtraction of proteins detected in the control from proteins detected in the respective treatment. The resulting lists were used as input for ClueGO analysis using the KEGG pathway database (Fig 4A). IL-1β-treated HUVEC displayed a clear pro-inflammatory signature with the NF-κB and the NOD signaling pathways being significantly enriched. Additionally, several infection-related pathways (Shigellosis, Salmonella infection, and Legionellosis) were found to be enriched, likely due to the overlap between these pathways and the NFκB response. These enrichments underline the inflammatory activation of IL-1β-treated HUVEC and are well in line with other studies [12,25,46].

VEGF stimulated HUVEC displayed an enrichment of the VEGF, ErbB and B-cell receptor signaling pathway, This enrichment pattern is mainly characterized by induction of MAP-kinases MAP2K1 and MAP2K2 as well as SHC1, underlining the pro-proliferation, pro-cell survival and anti-apoptotic action of VEGF. MAP2K2, however, was also found to be induced in IL-1β-treated HUVEC. We interpret these results in the way that MAP2K2 and not MAP2K1 is mainly responsible for overlapping effects in inflammatory and angiogenic (IL-1β and VEGF) activated HUVEC.

Several cancer-related pathways (Non small cell lung cancer, melanoma, glioma, chronic myeloid leukemia, renal cell carcinoma, central carbon metabolism in cancer and acute myeloid leukemia) were also found to be enriched, underlining the role of VEGF in these cancers. In the “acute myeloid leukemia” and the “central carbon metabolism in cancer pathways”, MAP2K2, JUP, and PFKM are induced by IL-1β as well. The induction of SRPs (SRP19, SRP54, and SRP72) and the resulting enrichment of the protein export pathway reflects the secretory activation of HUVEC by both IL-1β and VEGF.

Interestingly two pathways related to neurodegenerative disorders (Alzheimer's disease and Parkinsons's disease) and connected to oxidative phosphorylation were also found to be enriched. In this context, a group of NADH ubiquinone oxidoreductases (NDUFA7, NDUFA10, NDUFA13, NDUFB1, NDUFS1), as well as HTRA1 and POLR2H was expressed by VEGF stimulated HUVEC.

HTRA1 is a serine protease with a variety of targets. Intracellularly, it degrades TSC2 which leads to the activation of TSC2 downstream targets [47]. Extracellularly, HTRA1 is a regulator of the IGF and TGF-β signaling pathways via cleavage of IGF-binding proteins respectively TGF-β family members [48]. Thus induction of HTRA1 by VEGF might represent a regulatory crosslink between IGF or TGF-β and VEGF signaling. The induction of RNA polymerase II (POLR2H) reflects the increased transcriptional activity in activated HUVEC.

Although these pathways were assigned to the VEGF-cluster due to a stronger enrichment in VEGF-stimulated HUVEC, several of the NADH-ubiquinone oxidoreductase subunits were also found to be induced in IL-1β-treated HUVEC as well. All NDUs are members of the complex I, the first enzyme of the mitochondrial respiratory chain which catalyzes the transfer of electrons from NADH to coenzyme Q10 and is a powerful source of reactive oxygen species (ROS) [49]. ROS are known to serve as second messengers for VEGF, especially for EC migration [50]. On the other hand, function reducing mutations in these genes lead to an increased level of reactive oxygen species [51]. Thus, upregulation of NADH-ubiquinone oxidoreductases may not only reflect the activated state of the cells but also point towards a ROS protective role of these genes implying the activation of a ROS protective system during inflammation and angiogenesis.

At the crossroads between IL-1β and VEGF the “bladder cancer pathway” was found to be enriched. This enrichment was characterized on one side by induction of MAP2K1, MAP2K2 and CDKN2A in VEGF-stimulated HUVEC and MAP2K2, CXCL8, and CDKN2A in IL-1β stimulated HUVEC. The “bladder cancer pathway” is characterized by two branches, namely induction of VEGF and CXCL8 (IL-8) via the ErB signaling pathway and cell cycle activation via the MAPK-pathway. Members of both branches could be found to be expressed in both IL-1β and VEGF-activated HUVEC, pointing towards a possible crosstalk of IL-1β and VEGF activation via these pathways.

#### Nuclear extract

The biological context of proteins of the nucleic fraction was investigated using the gene ontology biological process database with parameters as described above (Fig 4B).

In both treatments, GO-terms related to vesicle targeting, vesicle coating and vesicle transport confirm the amplified secretory activity of cells. Additionally, microtubule-based transport was found to be enriched in VEGF-activated HUVEC. In IL-1β-treated HUVEC several general transcription factors (GTFH1 and GTH2H4) were found to be induced, likely due to the general transcriptional activation of the cell. This is reflected in the enrichment of GO-terms associated with replication. The proliferative state of VEGF-activated HUVEC reflects in the enrichment of GO-terms associated with cell proliferation (purine ribonucleoside monophosphate biosynthetic process, positive regulation of G1/S transition of mitotic cell cycle). Contrary to that, positive regulation of cell cycle arrest was found to be enriched in IL-1β-treated HUVEC. The enrichment of “apoptotic process” involved in morphogenesis confirms the role of VEGF in apoptosis protection.

#### Extracellular proteins

The biological context of detected extracellular proteins was investigated using the gene ontology biological process database with parameters as described above (Fig 4C). The signature of IL-1β-treated HUVEC was characterized by several interconnected groups of GO-terms associated with the inflammatory response, activation of cells of the immune system, regulation of chemotaxis, and phosphorylation of STAT. The protein synthetic and secretory activity is reflected in the enrichment of processes associated with transcriptional, protein transport and protein secretory activity. The specific pathway signature of VEGF-activated HUVEC was characterized by the enrichment of terms associated with ribonucleoside synthesis, activation of MAPKK activity and, interestingly, terms associated with antigen processing and presentation via MHC class II. Additionally “negative regulation of ERB signaling pathway” activity was found to be enriched. Recently it has been shown that IL-1β and VEGF activation signatures of HUVEC overlap on mRNA level to a considerable extent [12]. In our data, however, the overlap of the signatures was restricted to general catabolic processes, for instance, polysaccaride or carbohydrate catabolism, thus reflecting the activated state of the cells.

### Weighted protein co-expression network analysis of extracellular proteins

Based on the concept of network centrality, weighted protein co-expression analysis (WPCNA) allows the determination of potential key driver genes in a process, therefore we performed WPCNA on protein coexpression networks defined by selected GO-terms for untreated, IL-1β, and VEGF-activated HUVEC. for the individual GO-terms and calculated co-expression parameters. For each gene, we calculated the sum of connectivities as a measure of the centrality of the gene within the co-expression network and normalized the connectivity against the sum of connectivities within the module. A summary of connectivities is listed in S3 Table.

#### Angiogenesis

**Fig 5:**
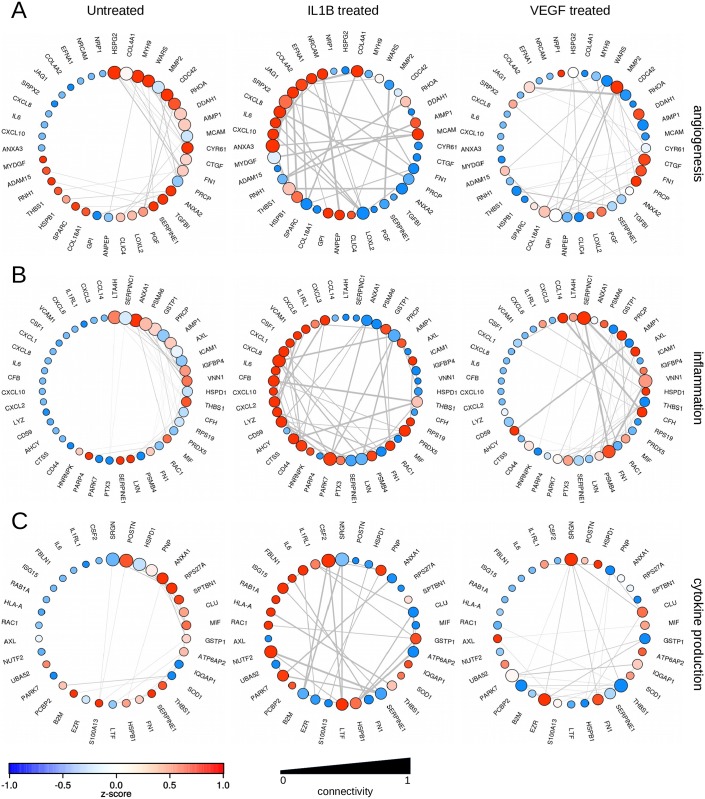
Co-expression networks of untreated HUVEC, HUVEC treated with 100ng/mL IL-1β and HUVEC treated with 100ng/mL VEGF. (A) GO Term GO:0001525 –angiogenesis (B) GO term GO:0006954 inflammatory response and GO term GO:0001816 cytokine production. Node size indicates the connectivity within the GO-term, node color the z-score normalized up-regulation (red) resp. down-regulation (blue). Edge width indicates the normalized strength of the co-expression. To facilitate comparison and highlight differences, genes within treatments are sorted according to intra-module connectivities in quiescent HUVEC.

The co-expression network of resting HUVEC within this module displays eight interconnected genes, HSPG2, COL4A1, MYH9, WARS, MMP2, CDC42, RHOA, and DDAH1 (connectivity > 0.75). Querying pathway commons shows that all five proteins are part of a closely interconnected interaction network containing 948 member genes, 4943 interactions associated with state change and 164 interactions associated with regulation of expression. The pathway commons network neighborhood analysis places all 8 potential hub proteins at positions with a dense network connectivity (i.e. displaying a large number of interactions with other genes), thus confirming their potential hub status (Fig 5a)

As a key component of the extracellular matrix, HSPG2 (Heparan Sulfate Proteoglycan 2) plays an essential role in multiple biological activities, encompassing maintenance of the endothelial barrier function, vascular homeostasis, and inhibition of smooth muscle cell proliferation. It is thus thought to help maintain vascular homeostasis. It also has a promoting effect on growth factor (e.g., FGF2) activity and may stimulate endothelial growth and regeneration [52].

MMP2 (Matrix metalloproteinase 2) plays a crucial in remodeling the vasculature, angiogenesis, and inflammation via its extracellular matrix-degrading activities. The C-terminal non-catalytic fragment of MMP2 displays anti-angiogenic properties and inhibits cell migration and cell adhesion to FGF2 and vitronectin [53]. Additionally, MMPs play an important role in the regulation of inflammatory and immune responses [54]. Taken together, the co-expression pattern of potential key drivers in the extracellular fraction of quiescent HUVEC points towards the existence of multiple checkpoints (HSPG2 upregulated, MMP2 downregulated) which act in concert to stabilize an antiinflammatory and antiangiogenic environment.

The co-expression pattern of IL-1β-treated HUVEC shifts towards a pro-inflammatory and proangiogenic signature with the potential key drivers being ANXA3, SRPX2, MYDGF, THBS1, LOXL2 and to a lesser extent CXCL8 as well as COL4A1 and COL4A2. The interactome neighborhood of these proteins consists of 536 members with 1450 state change controlling interactions and 209 expression controlling interactions. With regard to the expression of proteins, key drivers are antagonistically expressed when compared to quiescent HUVEC (e.g. HSPG2). This reversal of expression patterns reflects inflammatory activation. ANXA3 (Annexin-3) functions in the inhibition of phospholipase A2 and cleavage of inositol 1,2-cyclic phosphate to form inositol 1-phosphate. SRPX2 (Sushi-Repeat Containing Protein, X-Linked 2) acts as a ligand for the urokinase plasminogen activator surface receptor and acts as an inductor of endothelial cell migration and vascular network formation. It further increases phosphorylation levels of FAK and promotes cell proliferation by increasing the mitogenic activity of HGF [55]. COL4A1 interacts with extracellular matrix components such a perlecans, proteoglycans, and laminins. Both COL4A1 and A2 give rise to anti-angiogenic peptides (arresten from the c-terminus of COL4A1 and canstatin from the c-terminus of COL4A2), thus playing a role in the regulation of angiogenesis [56]. THBS1 (Thrombospondin 1) is an adhesive glycoprotein that mediates cell-to-cell and cell-to-matrix interactions. It binds bind to fibrinogen, fibronectin, laminin, type V collagen and integrins α-V/β-1 and plays a role in platelet aggregation and angiogenesis [57]. Together with COL4A1 and COL4A2, THPS is a member of the PI3K-Akt signaling superpathway and the Signaling by GPCR superpathway (http://www.genecards.org) [58].

Somewhat surprisingly, “classical” inflammation and angiogenesis associated cytokines and chemokines such as IL-6 or CXCL8 are far less connected within this co-expression network. These cytokines appear very early during the inflammatory response with mRNA detectable as early as 30 minutes after stimulation (see Fig 3D) [46]. It has been shown in lung epithelial cells, that expression of IL6 and CXCL8 continues to increase 16 hours after inflammatory stimulation with IL1b [59]. However, in our experimental setting (i.e. 24 hours incubation) the comparatively low network connectivity of IL-6, CXCL8 etc. may be indicative for a paracrine rather than autocrine/regulatory role in this context. Less obvious, but in terms of network connectivity well interconnected proteins such as ANXA3, MYDGF as well as THBS1 may serve as key regulators for angiogenesis processes.

The co-expression pattern of VEGF in term angiogenesis turned out to rest on only two genes, GPI and MMP2. Other known pro-angiogenic factors such as CTGF and collagens COL18A1 and COL4A2 display a high connectivity (0.7478, 0.7187 and 0.6372 respectively) but not high enough to be regarded as hub proteins. The interactome neighborhood consists of 268 proteins interconnected via 772 state changing interactions, 118 expression controlling interactions and 3 consecutive catalysis interactions. GPI is a glycolipid serving as an anchor for many key proteins posttranslational modification [60]. We interpret the key driver gene status in VEGF-activated HUVEC—as opposed to collagens and CTGF—in the way that posttranslational modification might play a key role in VEGF-mediated processes. Interestingly, the interaction network of GPI consists of two proteins (GNPDA1 and 2) and mainly of μRNAs which control GPI expression [61]. GNPDA1 and 2 are connected to GPI via a consecutive catalysis interaction, i.e. one protein produces the substrate of the other. Several μRNAs of the GPI-GNPDA1/2 network (e.g. MIMAT0000422) also control the expression of CTGF [61]. We interpret those data in a way that GPI and CTGF expression is at least partly co-regulated by μRNAs.

The expression pattern of the potential key driver proteins differs strikingly compared to quiescent as well as IL-1β activated HUVEC. As expected MMP2 is strongly overexpressed. Expression of GPI is in the middle between quiescent and IL-1β activated HUVEC and CTGF is again strongly overexpressed. Surprisingly, none of the inflammation markers found to be transiently upregulated in mRNA could be found in the secretome of VEGF-activated HUVEC [12]. COL4A1 and A2 are strongly underexpressed (COL4A2) or slightly overexpressed (COL4A1) after VEGF stimulation. Several angiogenesis-associated proteins like CTGF or FN1 are strongly overexpressed but display a low connectivity (0,685 resp. 0,596) that does not warrant classification as key drivers. Again we interpret these results in the direction that CTGF is an important paracrine rather than an autocrine factor.

#### Inflammatory response

The coexpression network within this module (GO:0006959) centers in quiescent HUVEC around LTA4H, SERPINC1, ANXA1, PSMA6, GSTP1, PRCP, AXL, and AIMP1. The interactome neighborhood is characterized by 256 proteins, 4876 state changing interactions and 65 expression controlling interactions. Most of these interactions, however, are concentrated in one extensive interaction network, centering around PSMA6, a protein that is essential for the processing of class I MHC peptides within the immunoproteasome [62]. Other ligands in this network include subunits of the NFκB complex and other proteins involved in immunologic/inflammatory processes (Fig 5b).

The other major interaction network centers around SERPINC, which inhibits thrombin and activated serine proteases [63]. The regulatory pattern we observed (downregulated in IL-1β activated HUVEC, strongly upregulated in VEGF-activated HUVEC) reflecting the role of HUVEC within the blood coagulation cascade. Prominent positioning as a potential key driver in both untreated and VEGF-treated HUVEC suggests a role in maintaining endothelial integrity as well as VEGF associated inflammation. A further part of the network forms connections with ANXA1, a membrane-localized protein displaying anti-inflammatory activity [64]. ANXA1 is strongly upregulated in quiescent HUVEC, downregulated in IL-1β activated HUVEC, a regulation pattern in line with its anti-inflammatory properties. The status as a driver gene in quiescent HUVEC may point towards a key role in preserving the resting state with regard to inflammatory activation. A connection to AXL via SRC may indicate a regulation via AXL-mediated activation of AKT kinases, thus enhancing cell survival by prevention of apoptosis [65]. Upregulation of LTA4H in combination with a downregulated AXL suggests that key signaling components of the inflammatory response are switched off in quiescent HUVEC to safeguard against inflammatory activation. The regulation pattern across conditions follows that of ANXA1 with the exception that AXL it is strongly upregulated in VEGF-activated HUVEC.

LTA4H is a pro-inflammatory cytokine responsible for the generation of the inflammation mediator leukotriene B4. LTA4H also possesses aminopeptidase activity. Snelgrove et al demonstrated that the aminopeptidase activity acts on PGP, a major propagator of neutrophil-driven inflammation [66]. In this scenario, PGP is degraded by LTA4H, leading to a facilitated resolution of inflammation.

Although only 3 proteins can be considered hub genes for this biologic function in IL-1β activated HUVEC (CXCL8, MAPK1/2, and PARK), the interactome neighborhood consists of 353 proteins interconnected via 711 state changing interactions and 153 expression controlling interactions. Within the interactome context, CXCL8 is centered in an extensive network with several additional hubs, namely GRK6, MAPK1/3, EHMT1/2 and IRS1/2. The network extension around IRS1 and 2 contains many cytokines and chemokines, complemented by GRK6 which interacts mainly with CCLs and CXCLs [67]. The network extension around MAPK1/3 is characterized by growth factors such as the FGFs and PDGF. The EHMTs link into a network of histones. PRCP is an activator of the cell matrix-associated prekallikrein. Via angiotensin II, one of the substrates of this enzyme, PRCP plays a role in regulating blood pressure and electrolyte balance [68]. The interactome around PARK7 forms an independent network associated with chaperoning against oxidative stress. Taken together these results demonstrate the key role of CXCL8 in mediating the inflammatory response. Interestingly, other cytokines such as IL6, CXCL1, CXCL6 are far less interconnected in terms of co-expression which again leads us to the conclusion that these proteins are paracrine rather than autocrine factors.

Three proteins fulfill the criteria of hub proteins in VEGF-activated HUVEC, namely VNN1, FN1, SERPINC1. The interactome neighborhood of these proteins consists of 227 proteins interconnected via 445 state changing interactions, 49 expression controlling interactions and 94 consecutive catalysis interactions. The neighborhood of SERPINC1 has been described above. VNN1 (Vanin 1) hydrolyzes specifically one of the carboamide linkages in D-pantetheine thus recycling pantothenic acid (vitamin B5) and releasing cysteamine, thus linking metabolic diseases and inflammation [69]. FN1 (Fibronectin 1) is involved in cell adhesion and migration processes including embryogenesis, wound healing, senescence [70], blood coagulation, host defense, and metastasis. It is also a member of the PI3K-Akt signaling pathway where it acts via binding to ITGA and ITGB. VEGF elicits a considerable pro-inflammatory response on mRNA level [12]. Whereas IL-1β induces a strong and lasting upregulation of these genes on mRNA level, VEGF-induced activation is transient, peaks between 120 and 150 minutes and returns to control levels after an incubation period of 360 minutes (Fig 3D). We could detect only 5 gene products induced by IL-1β and VEGF on mRNA level in the supernatant of IL-1β-treated HUVEC, but none in the supernatant of VEGF-treated HUVEC (CXCL1, 2 and 3, IL8 and VCAM1, all markers for inflammation). We interpret these findings the way that VEGF induces a short-lived inflammatory response in endothelial cells that may translate into a weak pro-inflammatory protein expression profile that has to be amplified by other factors to persist.

#### Cytokine production

The coexpression network within this module (GO:0001816) centers in quiescent HUVEC around SRGN, POSTN, and HSPD1. The interaction network consists of only 88 proteins with 142 state change controlling interactions and 51 expression controlling interactions. The interaction network centers around HSPD1, a member of the chaperonin family. Although it is a mitochondrial protein it may also serve as a signaling molecule in the innate immune system. POSTN, as well as SRGN, are more peripherally located. However, all three share a common regulatory element, namely MAZ. Interestingly, HSDP1, as well as SRGN, is downregulated in IL-1β activated HUVEC but upregulated in VEGF-activated HUVEC. Together with the fact that the ClueGO pathway analysis revealed several immunology pathways to be enriched in VEGF-activated HUVEC (antigen processing and presentation), this might point towards a role of VEGF in immunological processes (Fig 5c)

For IL-1β activated HUVEC SRGN, CSF2, NUTF2, and LTF represent potential hub proteins in this module. The corresponding interaction network consists of 344 proteins with 810 state changing interactions and 10 expression controlling interactions. The interaction network is characterized by a large KRAS, HRAS, and NRAS segment strongly interconnected with CSF2. CSF2 is a member of the Interleukin-3, 5 and GM-CSF signaling superpathway. We interpret these observations in the sense that CSF2 plays a key driver role in IL-1β mediated cytokine production.

Finally, for VEGF-activated HUVEC THBS1, SRGN, PARK7, and EZR were identified as potential hub proteins. The interaction network consists of 115 proteins connected by 110 state change controlling interactions and 48 expression controlling interactions. THBS1 and EZR form the center of the network which extends via POFUT2 and B3GLCT into a densely interconnected network dominated by ADAMTs. The network centered by EZR is characterized by MAP kinases (e.g. MAP3K8, MAP4K4) and growth factor receptors (e.g. EGFR). EZR is a membrane protein functioning in surface structure adhesion and cell migration. However, it may also play a role in transducing growth-related signals.

PARK7 again is the center of a small network with chaperone function. The function of SRGN is described above.

#### Concluding remarks

Although endothelial cells have been subjected to extensive research on mRNA and single protein level with regard to angiogenic and/or inflammatory activation, up to now results encompassing the proteome are sparse. This study is, therefore, novel in several aspects.

First, this study represents the first report directly comparing quiescent, inflammatory and angiogenic activated HUVEC on proteomics level.

Second, the fractionation into cytoplasm, nuclei, and the extracellular space allows for a more detailed picture of the activation processes in major cellular compartments. Especially the secretome of HUVEC has not been investigated to this extend so far.

Finally, in this study we apply for the first time state-of-the-art computational biology methods such as Weighted Gene co-expression Analysis and in-depth pathway analysis methods to proteomics data based on cellular fractions rather than total cell or tissue extracts.

In conclusion, this study reports a reference proteome for the cellular, nucleic, and extracellular fraction of quiescent, inflammatory, and angiogenic activated HUVEC. It also identifies novel potential hub genes in these processes and provides a framework for the computational biology-based analysis of proteomics data. As a proof of concept validation of the identified hub genes and their impact on endothelial function could be done in further experiments using approaches such as RNAi.

## Supporting information

S1 Table Expression data of proteomics experiments (mean normalized counts per million). Summarized and normalized protein spectra counts in cpm, grouped by treatment and protein.
(XLS)

S2 Table ClueGO GO-term analysis results. Summarized ClueGO GO-Term results. GOID: GO Identification number, GOTerm: GO Term name, c: associated Genes found, % associated Genes: percentage of associated genes found, Term Pvalue: uncorrected p-value for the respective GO-term, Term PValue Corrected with Benjamini-Hochberg: corrected p-value for the respective GO-Term, Associated Genes Found: list of associated genes found., Genes IL-1beta treated HUVEC and %Genes IL-1beta treated HUVEC: list of genes found in IL-1beta treated HUVEC and percentage of the total genes of the GO-Term, Genes VEGF treated HUVEC and %Genes VEGF treated HUVEC: the same for VEGF treated HUVEC.
(XLS)

S3 Table Normalized modular connectivities for selected GO-terms. Summarized modular connectivities of modules angiogenesis, cytokine production and inflammatory response.
(XLS)
